# Glutathione affinity chromatography for the scalable purification of an oncolytic virus immunotherapy from microcarrier cell culture

**DOI:** 10.3389/fbioe.2023.1193454

**Published:** 2023-06-15

**Authors:** Andrew R. Swartz, Yvonne Shieh, Amanda Gulasarian, Erik Curtis, Carl F. Hofmann, Jack B. Baker, Neil Templeton, Jessica W. Olson

**Affiliations:** ^1^ Process Research and Development, Merck & Co., Inc., Rahway, NJ, United States; ^2^ Analytical Research and Development, Merck & Co., Inc., Rahway, NJ, United States

**Keywords:** oncolytic virus, glutathione, affinity chromatography, purification, empty full capsid separation, microcarrier cell culture, infection temperature, bioprocess scale-up

## Abstract

Therapeutic viral vectors are an emerging technology with several clinical applications in gene therapy, vaccines, and immunotherapy. Increased demand has required the redevelopment of conventional, low-throughput cell culture and purification manufacturing methods such as static cell stacks and ultracentrifugation. In this work, scalable methods were investigated for the manufacture of an oncolytic virus immunotherapy application consisting of a prototype strain of coxsackievirus A21 (CVA21) produced in adherent MRC-5 cells. Cell culture was established in stirred-tank microcarrier bioreactors, and an efficient affinity chromatography method was developed for the purification of harvested CVA21 through binding of the viral capsids to an immobilized glutathione (GSH) ligand. Bioreactor temperature during infection was investigated to maximize titer, and a decrease in temperature from 37°C to 34°C yielded a two–three-fold increase in infectivity. After purification of the 34°C harvests, the GSH affinity chromatography elution not only maintained a >two-fold increase in infectivity and viral genomes but also increased the proportion of empty capsids compared to 37°C harvests. Using material generated from both infection temperature setpoints, chromatographic parameters and mobile phase compositions were studied at the laboratory scale to maximize infectious particle yields and cell culture impurity clearance. Empty capsids that co-eluted with full capsids from 34°C infection temperature harvests were poorly resolved across the conditions tested, but subsequent polishing anion exchange and cation exchange chromatography steps were developed to clear residual empty capsids and other impurities. Oncolytic CVA21 production was scaled-up 75-fold from the laboratory scale and demonstrated across seven batches in 250 L single-use microcarrier bioreactors and purified with customized, prepacked, single-use 1.5 L GSH affinity chromatography columns. The large-scale bioreactors controlled at 34°C during infection maintained a three-fold increase in productivity in the GSH elution, and excellent clearance of host cell and media impurities was observed across all batches. This study presents a robust method for the manufacture of an oncolytic virus immunotherapy application that may be implemented for the scalable production of other viruses and viral vectors which interact with glutathione.

## 1 Introduction

Viral vectors are modified viruses that deliver therapeutic genetic material to target cells and are an emerging platform for gene therapy (Bulcha et al., 2021), vaccines (Travieso et al., 2022), and oncolytic virotherapy applications (Lundstrom, 2018). During viral vector cell culture production, empty capsids lacking genetic material are also generated and represent a key product-related impurity due to potential off-target immune responses (Wright, 2014; Klasse, 2015). The separation of empty capsids from genome-containing (full) particles poses a significant challenge to purification processes because of their similar size and surface properties (Dickerson et al., 2021). Traditional viral vector manufacturing methods utilize poorly scalable gradient ultracentrifugation to clear empty capsids from full capsids with higher particle density (Hermens et al., 1999; Dormond et al., 2010; Ichim and Wells, 2011). Advancement in chromatographic separations has enabled more robust and scalable purification processes for some viral vectors. For example, current adeno-associated virus (AAV) vector manufacturing processes replaced ultracentrifugation with affinity and ion exchange chromatography for host cell impurity and empty capsid clearance (Nass et al., 2018; Adams et al., 2020). However, economically viable large-scale affinity chromatography has not yet been widely implemented for other viral vectors due to challenges of affinity ligand design (Singh and Heldt, 2022).

A prototype strain of coxsackievirus A21 (CVA21), a non-enveloped enterovirus in the *Picornaviridae* family, is undergoing clinical evaluation as an oncolytic virus immunotherapy application for the treatment of several types of cancers (Bradley et al., 2014; Annels et al., 2019; Andtbacka et al., 2021). Similar to conventional viral vector production (Segura et al., 2011), early batches of CVA21 were purified in small quantities from static MRC-5 cell culture by gradient ultracentrifugation for clearance of upstream impurities including bovine serum albumin (BSA), host cell DNA (HC-DNA), and empty viral capsids (Swartz et al., 2022). Scalable upstream and downstream CVA21 production technologies were investigated including microcarrier stirred-tank bioreactors and affinity chromatography to replace static cell culture and ultracentrifugation (Swartz et al., 2022). The growth of adherent cells on suspended microcarrier beads in stirred-tank bioreactors has been demonstrated for the production of other viruses (Wu et al., 2004; Trabelsi et al., 2012), but there was no known commercially available affinity chromatography ligand for CVA21. Heparin-based pseudo-affinity chromatography (Zhao et al., 2019) has been evaluated for other picornaviruses with limited selectivity and capacity due to the many host cell heparin-binding proteins present in cell culture harvests (Gu et al., 2018; Park et al., 2020).

Based on reports that enteroviruses interact with intracellular antioxidant glutathione (GSH) during capsid assembly in infected cells (Ma et al., 2014; Thibaut et al., 2014; Duyvesteyn et al., 2020), we developed a selective CVA21 affinity chromatography method by repurposing commercially available immobilized GSH resin originally intended for glutathione S-transferase (GST)-tagged recombinant protein purification (Swartz et al., 2022). Infectious, genome-containing capsids bound and eluted with high yield from all clarified harvests tested, but non-infectious, empty capsid binding to the GSH ligand was found to depend on the cell culture conditions during infection. A small-scale microcarrier bioreactor MRC-5 cell culture process was established and initially maintained at 37°C during both cell growth and viral infection stages. The empty capsids present in these clarified harvests flowed through during the GSH affinity chromatography loading step, resulting in high purity of full capsids in the elution. However, empty capsids that were harvested from bioreactors controlled at sub-physiological temperature during infection bound to the GSH ligand and co-eluted with full capsids.

The objective of this work was to establish a robust and scalable purification method of CVA21 with high infectivity yield and purity that can accommodate variable cell culture conditions. We first optimized the bioreactor cell culture temperature during CVA21 infection that maximized viral titer and evaluated the impact on downstream purification. The GSH affinity chromatography empty capsid clearance of the virus harvested from the most productive infection temperature of 34°C was compared to the original infection setpoint of 37°C. Second, chromatographic parameters including loading residence time, binding capacity, and wash and elution buffers (Supplementary Figure S1) were investigated to increase the purity of infectious particles from cell culture impurities and empty capsids. Third, the microcarrier cell culture and optimized GSH affinity chromatography methods were scaled-up to a 250 L process scale, and CVA21 harvests produced at 34°C during infection were compared to the 37°C infection condition. Collectively, these studies enabled an efficient method for CVA21 manufacture that may be implemented as a production platform for enteroviral vectors.

## 2 Materials and methods

### 2.1 Materials and buffers

Human diploid lung fibroblast MRC-5 cells were passaged from the European Collection of Authenticated Cell Cultures (#05072101). Virus stocks containing a prototype strain of CVA21 were derived from the Kuykendall strain obtained from the ATCC (VR-850). Cell culture was performed in Gibco William’s Medium E, Modified (WMEM) (Cat#: 12551032, Thermo Fisher Scientific, Waltham, MA) media supplemented with L-glutamine (Cat#: G7513, Sigma Aldrich, St. Louis, MO), and bovine calf serum (BCS) (Cat#: SH30073.04HI, Hyclone, Logan, UT). Media used for microcarriers were supplemented with poloxamer 188 (Cat#: P5556, Sigma-Aldrich, St. Louis, MO). In some cases, the harvest cell culture was treated with Benzonase^®^ endonuclease (Cat#: 101697, Merck KGaA, Darmstadt, Germany). Phosphate-buffered saline (PBS) (Thermo Fisher Scientific) was used to flush and chase clarification filters. All chromatography buffers were prepared in Milli-Q Ultrapure water (MilliporeSigma, Burlington, MA) with 10–15 mM Tris-HCl (Thermo Fisher Scientific) and 0.005% Polysorbate 80 (PS80) (Croda, Snaith, United Kingdom), and the indicated sodium chloride (NaCl) (Teknova, Hollister, CA) concentration and pH were adjusted with either 50% sodium hydroxide (NaOH) (Thermo Fisher Scientific) or 37% hydrochloric acid (Thermo Fisher Scientific). All GSH wash 2, elution, and strip buffers contained 1 mM dithiothreitol (DTT) (Thermo Fisher Scientific). GSH elution and strip buffers contained the indicated reduced L-glutathione (GSH) (Thermo Fisher Scientific) concentration.

### 2.2 Laboratory-scale bioreactor cell culture

At the laboratory scale, MRC-5 cells were cultured in 3 L glass bioreactors with a BioFlo 320 controller (Cat#: 1379962911, Eppendorf, Hamburg, Germany) on 1 g/L Cytodex 1 microcarriers (Cat#: 17548702, Cytiva, Marlborough, MA) or in iCELLis Nano fixed-bed bioreactors (Pall Corporation, Port Washington, NY) with a 10 cm bed height with WMEM and 10% BCS controlled at a target temperature of 37°C and pH of 7.2 as described previously (Swartz et al., 2022). Ambr^®^250 HT reactors (Sartorius AG, Göttingen, Germany) with 1 g/L Cytodex 1 microcarriers were operated for oncolytic virus production similar to a published method (Brown et al., 2023). When cells reached >90% confluency, the 3 L glass and Ambr^®^250 HT bioreactors underwent an 80% medium exchange into WMEM without serum for a final concentration of 2% (v/v) BCS. For iCELLis Nano bioreactors, a 100% medium exchange into WMEM without serum was performed. Immediately prior to infection, the temperature setpoint was either maintained at 37°C or changed to a range of temperatures between 31°C and 38.5°C across multiple experiments with each condition performed in duplicate (Table 1; Supplementary Figure S2C). Cells were infected with a CVA21 virus stock at a MOI of 0.05–0.5 pfu/viable cell, and the cell lysate was harvested 4–5 days post-infection at a cytopathic effect (CPE) of >90%. Sample retains were taken at various time points post-infection and stored at −70°C until analysis. The lysate was clarified through a series of 60 µm Clarisolve 60HX (Cat#: CS60HX01L3, MilliporeSigma) and 1.2 µm Sartopure GF+ (Cat#: 5555303 PV, Sartorius AG) filters to remove microcarriers and large cell debris to generate the clarified bulk (CB). In some cases, the CB was treated with a Benzonase^®^ endonuclease. The CB was frozen and stored at −70°C and then thawed and equilibrated to ambient temperature before use. CBs that were produced from 3 L microcarrier bioreactors controlled at 37°C or 34°C during infection or from iCELLis reactors controlled at 37°C during infection were utilized for laboratory-scale GSH affinity chromatography experiments.

**TABLE 1:** Temperature during infection and the bioreactor platform across three sets of experiments. Each condition was performed in duplicate.

Experiment	Infection temperature (°C)	Bioreactor platform
1	34, 35.5, 37, and 38.5	3L Bioreactor
2	32.5, 34, and 37	3L Bioreactor
3	31, 32.5, 34, and 37	Ambr 250 HT

### 2.3 Laboratory-scale glutathione affinity chromatography

Unless otherwise indicated, laboratory-scale GSH affinity chromatography was performed with GSTPrep FF 16/10 columns packed with GSH Sepharose 4 FF resin (Cat#: 28936550, Cytiva) run at a 6 min residence time using an AKTA Pure 150 M FPLC system (Cytiva) with UNICORN system control software (Cytiva). All chromatography buffers contained 10–15 mM Tris-HCl buffer at pH 8.0 or the indicated pH and 0.005% PS80. The GSH column was equilibrated with 5-column volume (CV) buffer containing 150 mM NaCl (Equil Buffer). CB was directly loaded without solution adjustment at 150–200-CVs followed by 8–10-CV of wash 1 buffer at 300–400 mM NaCl or the indicated NaCl concentration gradient and 4–5-CVs of wash 2 buffer at 75–100 mM NaCl and 1 mM DTT. Elution was performed with 4–5-CVs of elution buffer containing 75–150 mM NaCl, 1 mM DTT, and 1 mM GSH or the indicated NaCl or GSH concentration gradient. The column was stripped with 4–5-CVs of a buffer containing 1M NaCl, 1 mM DTT, and 10 mM GSH and was regenerated (regen) with a 0.1 M NaOH and 1 M NaCl solution. The chromatography step or gradient fractions’ flow-through (FT), wash, elution, strip, and regen were collected using the AKTA system outlet valve or fraction collector. Samples were frozen and stored at < −70°C until analysis. Chromatography sample analytical yields were calculated by dividing the analyte mass in the chromatography fraction by the loaded analyte mass in the CB.

Residence time and GSH resin-type experiments were initially evaluated on the 1 mL column scale with 37°C infection temperature CB using prepacked HiTrap Sepharose 4 FF (GSTrap FF, Cat#: 17513002, Cytiva) and Sepharose 4B (GSTrap 4B, Cat #: 29048609, Cytiva) columns and Pierce Glutathione columns (Cat#: 16109, Thermo Fisher Scientific). Columns were run at 0.5, 1.5, 3, and 6 min residence times using the same method, and the GSH elution infectivity yield was evaluated. The impact of residence time on the elution yield was evaluated using the CB from both 37°C and 34°C infection temperatures using 20 mL prepacked GSTPrep FF 16/10 columns across residence times of 2–12 min. Chromatography breakthrough experiments were performed using the CB from 34°C infection temperature and purified CVA21 diluted in GSH Equil buffer to the same viral particle concentration as the CB. The purified CVA21 sample was generated by GSH and polishing chromatography (see Section 2.5) and contained high-purity full capsids. Samples were loaded to a 20-mL GSH column at the indicated viral particle (by anti-VP1 CE Western) or volumetric loading, and breakthrough was calculated by dividing the FT analyte concentration by the loaded analyte concentration in the CB.

### 2.4 Scaled-up bioreactor cell culture and GSH affinity chromatography

The microcarrier bioreactor, clarification, and GSH affinity chromatography methods were scaled-up and executed using 100 and 250 L bioreactor volume in seven batches (Table 2). Cell culture was conducted in sterile 2 × 50 L or 250 L HyPerforma SUBs (Thermo Fisher Scientific) using a pitched-blade impeller in a 5:1 turndown ratio configuration using CX5-14 or Aegis 5-14 film (Thermo Fisher Scientific). Both impellers were upsized and sourced from a 100 L and 500 L bioreactor, respectively. MRC-5 cells were cultured on 1 g/L Cytodex-1 microcarriers in WMEM supplemented with 10% BCS controlled at a target temperature of 37°C and pH of 7.2. Prior to infection, the temperature setpoint was either maintained at 37°C (batches 1–3) or changed to 34°C (batches 4–7). Cells were infected with a CVA21 virus stock at a MOI of 0.05 pfu/viable cell, and the cell lysate was harvested approximately 4 days post-infection. The lysate was clarified by serial filtration through 1 or 2 × 0.55 m^2^ 60 µm Clarisolve 60HX pods (Cat#: CS60 H × 05 F1-X, MilliporeSigma) and 0.4 m^2^ or 0.8 m^2^ 1.2 µm Sartopure GF + Maxicap cartridges (Cat#: 5557303P1, Sartorius AG) to generate CB. In the indicated batches, the harvest was treated with Benzonase^®^ endonuclease. GSH affinity chromatography was performed with a custom 0.8 L OPUS 10 or 1.5 L OPUS 14 single-use column (Repligen, Waltham, MA), prepacked with Glutathione Sepharose 4 FF resin (Cat#: 17513203, Cytiva), using an AKTA ready single-use system and low-flow kit (Cytiva) with UNICORN system control software (Cytiva). The column was equilibrated with PBS, and 100–160 CVs of CB were loaded to the column with a 6 min residence time. The column was washed at a 4 min residence time with 8 CVs of wash 1 buffer at 400 mM NaCl, pH 8.0, conditioned with 4 CVs of wash 2 buffer at 75 mM NaCl, pH 8.0, and eluted with elution buffer at 75 mM NaCl and 1 mM GSH, pH 8.0. The column was stripped and regenerated similar to laboratory-scale experiments. Batch productivity was calculated by dividing the relative infectivity units in the GSH elution by the processed volume of the cell culture. For batches 6 and 7, the CB was also loaded to 20 mL GSH columns to evaluate scalability.

**TABLE 2:** Description of large-scale CVA21 cell culture in microcarrier single-use bioreactors and purification by glutathione affinity chromatography. Chromatography elution infectivity yield from clarified bulk and residual host cell DNA and bovine serum albumin concentrations shown. *Extrapolated HC-DNA concentration indicated if below the lower limit of quantitation, except when indistinguishable from no template controls.

Batch	1	2	3	4	5	6	7
Bioreactor vessel	2 × 50 L SUB	250 L SUB	250 L SUB	250 L SUB	250 L SUB	250 L SUB	250 L SUB
Infection temperature	37°C	37°C	37°C	34°C	34°C	34°C	34°C
Processed CB volume	80 L	100 L	200 L	220 L	240 L	240 L	240 L
Endonuclease treatment	Yes	Yes	No	No	No	No	No
Infectivity yield	88%	101%	97%	85%	86%	92%	94%
HC-DNA (ng/mL)*	0.017	0.021	<0.020	0.013	0.162	0.064	0.036
BSA (ng/mL)	11.0	13.5	85.3	41.3	37.9	42.8	15.0

### 2.5 Ion-exchange polishing chromatography of the GSH elution

To clear residual cell culture impurities from the GSH elution product, viral flow-through anion exchange (AEX) polishing chromatography was performed using 5 mL POROS 50 HQ GoPure columns (Cat#: A36639, Thermo Fisher Scientific) using the AKTA Pure 150 M and UNICORN control system. The column was equilibrated with AEX Equil buffer (15 mM Tris, 75 mM NaCl, and 0005% PS80, pH 8.0), and the GSH elution was directly loaded without solution adjustment. The AEX column was stripped using AEX Equil buffer with 300 mM and 1 M NaCl. The AEX FT sample was adjusted to pH 4 with a sodium citrate buffer containing a final concentration of 400 mM NaCl and loaded to a cation exchange (CEX) 5-mL POROS 50 HS GoPure column (Cat#: A36637, Thermo Fisher Scientific) to clear residual empty capsids present in the GSH elution from cell culture harvests produced at 34°C during infection as described previously (Konstantinidis et al., 2022). Full capsids were eluted from the CEX column with an 800 mM NaCl buffer.

### 2.6 Analytical Methods

#### 2.6.1 Relative infectivity assay

CVA21 sample relative infectivity was quantified by integrated high-throughput viral imaging of infectivity assay in SK-MEL-28 cells passaged from the American Type Culture Collection (ATCC, HTB-72) as described previously (Swartz et al., 2022). Here, 384-well poly-D-lysine cell plates (Corning, NY, Corning) seeded with SK-MEL-28 cells were infected with a pre-diluted CVA21 reference standard and test samples, followed by an 8 h incubation. The cells were fixed using a formaldehyde solution; then, immunostaining was performed using Hoechst 33342 nuclear DNA stain (Thermo Fisher Scientific), a polyclonal anti-CVA21 rabbit capture antibody, and an Alexa Fluor^®^ 488 AffiniPure Donkey Anti-Rabbit detection antibody (Jackson ImmunoResearch, West Grove, PA). Percent infected cells were quantified using Gen5 software (Agilent, Santa Clara, CA), and the data were fit to a four-parameter logistic model yielding an EC50 value. The % relative infectivity of a test sample was determined by dividing the EC50 value of a test sample by the EC50 value of a reference standard.

#### 2.6.2 Sodium dodecyl sulfate–polyacrylamide gel electrophoresis

Sodium dodecyl sulfate–polyacrylamide gel electrophoresis (SDS-PAGE) was used to detect viral and impurity protein bands. CB samples were pre-diluted 100x in water, while GSH chromatography fractions were not pre-diluted. The samples were mixed with 4x loading dye and 10x reducing agent (BioRad, Hercules, CA), heated at 70°C for 10 min, loaded in 15-well 12% acrylamide Bis-Tris NuPAGE gels (Thermo Fisher Scientific) along with a Mark12 standard (Thermo Fisher Scientific), and run at 200 V for 60 min using an Invitrogen gel box and power supply system (Thermo Fisher Scientific). Gels were stained using a Pierce Silver Stain kit (Thermo Fisher Scientific) and imaged using a gel imager (BioRad). CVA21 bands (VP0, VP1, VP2, VP3, and RNA) were identified based on their theoretical molecular weight (MW), and the detection of VP0 was used as a marker for empty capsids.

#### 2.6.3 Quantitative capillary electrophoresis Western blot

Capillary electrophoresis (CE) Western blot was performed using a Protein Simple Jess system (Protein Simple, Santa Clara, CA) and a 12–230 kDa 25-capillary Jess separation kit (SM-W004) based on a published procedure (Gillespie et al., 2022). Samples were prepared following the manufacturer’s instructions using the EZ Standard Pack (PS-ST01EZ) and anti-rabbit detection module (DM-001). Primary detection polyclonal antibodies anti-VP1 (Cat#: Q01161902, LifeTein, Somerset, NJ) and anti-VP4 (Cat#: Q01161904, LifeTein) were utilized at a concentration of 20 μg/mL. CE Western blot and sample analysis were performed following the default chemiluminescence assay protocol in Compass software Simple Western (Protein Simple). For VP1 total particle quantitation, anti-VP1 Western chemiluminescence peak areas were calibrated against a VP1 fragment (N-term 1–76, LifeTein) standard with a defined particle concentration. Sample VP0 and VP4 content was quantified using the anti-VP4 Western blot to detect both VP0 and VP4 peak areas relative to a reference standard. The empty-to-full capsid ratio was estimated using a relative ratio of VP0 to VP4 peak areas to a reference standard that contained a mixture of empty and full capsids.

#### 2.6.4 Reversed-phase ultra-performance liquid chromatography

A viral protein reversed-phase assay (RP-UPLC) was performed using a BioResolve RP mAb Polyphenyl, 450A, 2.7 um column (Cat#: 186008945, Waters, Milford, MA) operated at a flow rate of 0.4 mL/min at 80°C on an ACQUITY UPLC System (Waters), following a published method (Deng et al., 2022). CVA21 capsid proteins VP0, VP1, VP2, VP3, and VP4 were separated using an acetonitrile (ACN) (Thermo Fisher Scientific) gradient from 25%–80% ACN with 0.1% trifluoroacetic acid (TFA) (Thermo Fisher Scientific) and detected by intrinsic fluorescence (FLR detector) using excitation at 280 nm and emission at 352 nm. VP0 (empty only) and VP2 (full only) FLR peak areas were used to estimate % empty capsid by dividing the VP0 peak area by the sum of the VP0 and VP2 peak areas.

#### 2.6.5 Quantitative polymerase chain reaction assays

Viral RNA genomes and HC-DNA were quantified by RT-qPCR and qPCR assays, respectively, using the TaqMan^®^ Fast Virus 1-Step Master Mix (Thermo Fisher Scientific) on ABI 7900HT, ViiA 7, QuantStudio 5, or QuantStudio 7 Flex instruments (Life Technologies/Thermo Fisher Scientific). The samples were lysed using proteinase K and SDS at 37°C for 30 min, followed by nucleic acid extraction (Moore and Dowhan, 2002). Then, the sample lysate was mixed with phenol:chloroform:isoamyl alcohol, followed by phase separation. Nucleic acids were precipitated from the aqueous phase using sodium acetate and isopropanol, pelleted, and then washed with ethanol. The pellets were dried in an oven at 37°C and then resuspended in nuclease-free water. The extracted nucleic acids were used for both assays. In some cases, samples were pre-diluted in PBS prior to extraction, and the LOQ was adjusted accordingly. Extrapolated values were reported when the test result was below the LOQ (Table 2).

The viral genome RT-qPCR assay used a TaqMan^®^ MGB probe and primers designed against GenBank AF465515.1 and targeted a 101-nucleotide sequence in the CVA21 VP1 gene. The genome copy number was determined by interpolation against a standard curve of a synthetic RNA (Integrated DNA Technologies, Coralville, IA) containing the VP1 gene target region, ranging from 1E+11 to 1E+07 copies/mL. The HC-DNA qPCR assay used a TaqMan^®^ MGB probe and primers designed against RefSeq. NM_001164835.1 and targeted a 64-nucleotide sequence in the 3’ conserved region of the human LINE-1 transposase domain. The HC-DNA concentration was determined by interpolation against a standard curve of purified MRC-5 DNA, ranging from 2E+02 to 2E-02 ng DNA/mL. For both assays, each sample was measured in triplicate wells, and the average of the triplicate measurements is reported.

#### 2.6.6 BSA enzyme-linked immunosorbent assay

The BSA concentration of the GSH elution was quantified by using a commercially available BSA enzyme-linked immunosorbent assay (ELISA) kit (Cat#: F030, Cygnus Technologies, Southport, NC) following the manufacturer’s instructions and supplied reagents. All GSH elution samples were pre-diluted 10x in a sample diluent, and other samples targeted a dilution into the range of the standard curve. A SpectraMax M5 plate reader (Molecular Devices, San Jose, CA) was used to measure the absorbance at 450 nm. The BSA concentration was determined by fitting sample absorbance to the standard curve of pure BSA prepared from 32 to 0.5 ng/mL.

## 3 Results

### 3.1 Optimization of microcarrier bioreactor cell culture temperature during infection

**FIGURE 1:**
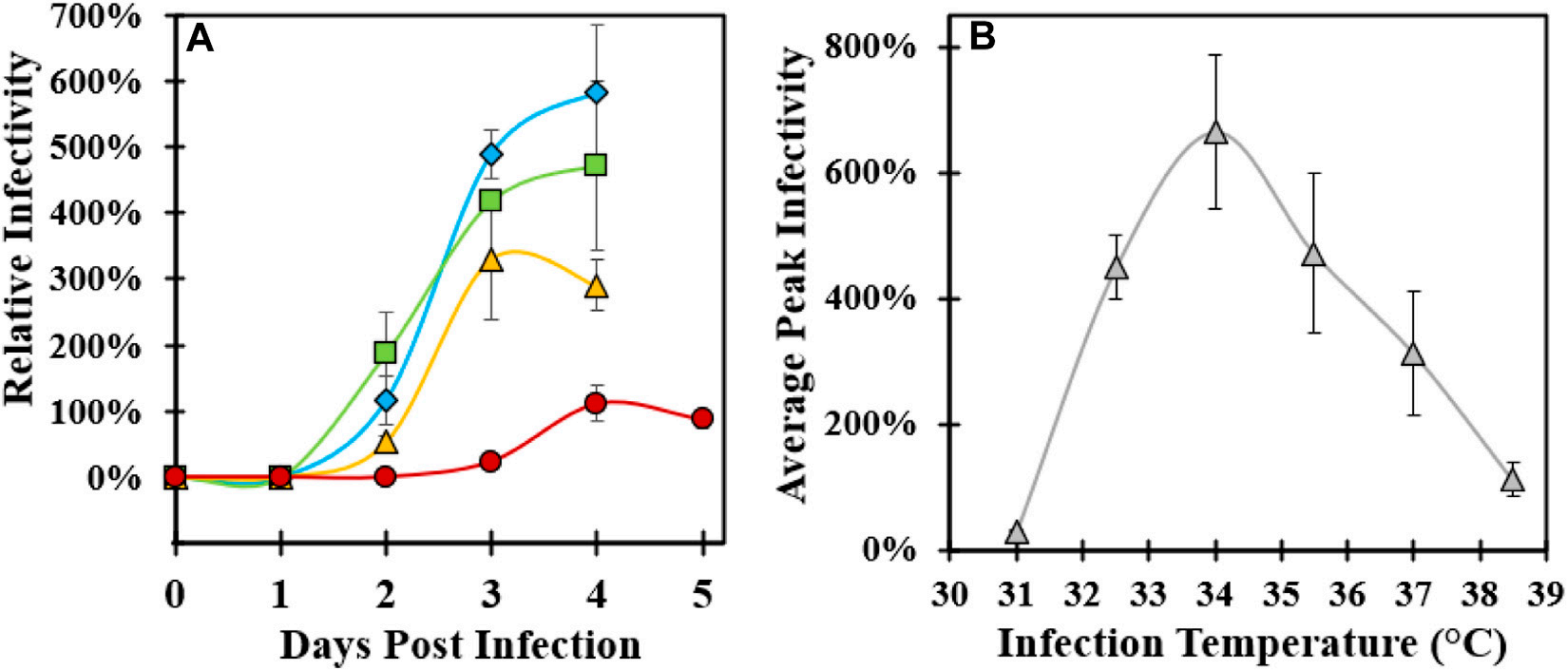
Optimization of microcarrier bioreactor temperature during CVA21 infection. **(A)** Relative infectivity sampled across 4–5 days post-infection controlled at 34°C (blue diamond), 35.5°C (green square), 37°C (yellow triangle), and 38.5°C (red circle). Error bars represent one standard deviation of an average of two replicate conditions. **(B)** Average peak relative infectivity, defined as the highest sample infectivity across the 4–5-day bioreactor infection phase, as a function of infection temperature across three experiments. Error bars represent one standard deviation of an average at each temperature condition. See Supplementary Figure S2C for a description of experimental replicates.

Cell culture temperature during infection was investigated to maximize the infectious CVA21 titer relative to the original infection temperature setpoint of 37°C. In each of the three experiments using 3 L and Ambr 250 microcarrier bioreactor systems, reactors were planted with the same preparation of MRC-5 cells and media with consistent growth conditions and media exchange. Immediately prior to infection, each bioreactor temperature was changed to a specified setpoint in the range of 31°C–38.5°C and maintained for 4–5 days post-infection with each condition run in duplicate (Table 1). In experiment 1, the peak virus infectivity increased with lower infection temperature with an approximate two-fold increase in productivity at 34°C relative to 37°C (Figure 1A). Decreased viral titer and slower infection kinetics were observed at 38.5°C compared to 37°C. To verify these results, lower infection temperature was evaluated in two subsequent experiments (experiments 2 and 3) that yielded nearly three-fold higher titer at 34°C infection temperature than the baseline 37°C condition (Supplementary Figures S2A, B). The peak infectivity ranged from 3–4 days post-infection, and the average peak infectivity was calculated at each temperature setpoint across the three experiments (Supplementary Figure S2C). The 34°C condition was confirmed to be the optimal infection temperature, and viral infectivity decreased below 34°C with almost no detectable infectious virus at 31°C (Figure 1B).

### 3.2 Impact of cell culture infection temperature on downstream purification

**FIGURE 2:**
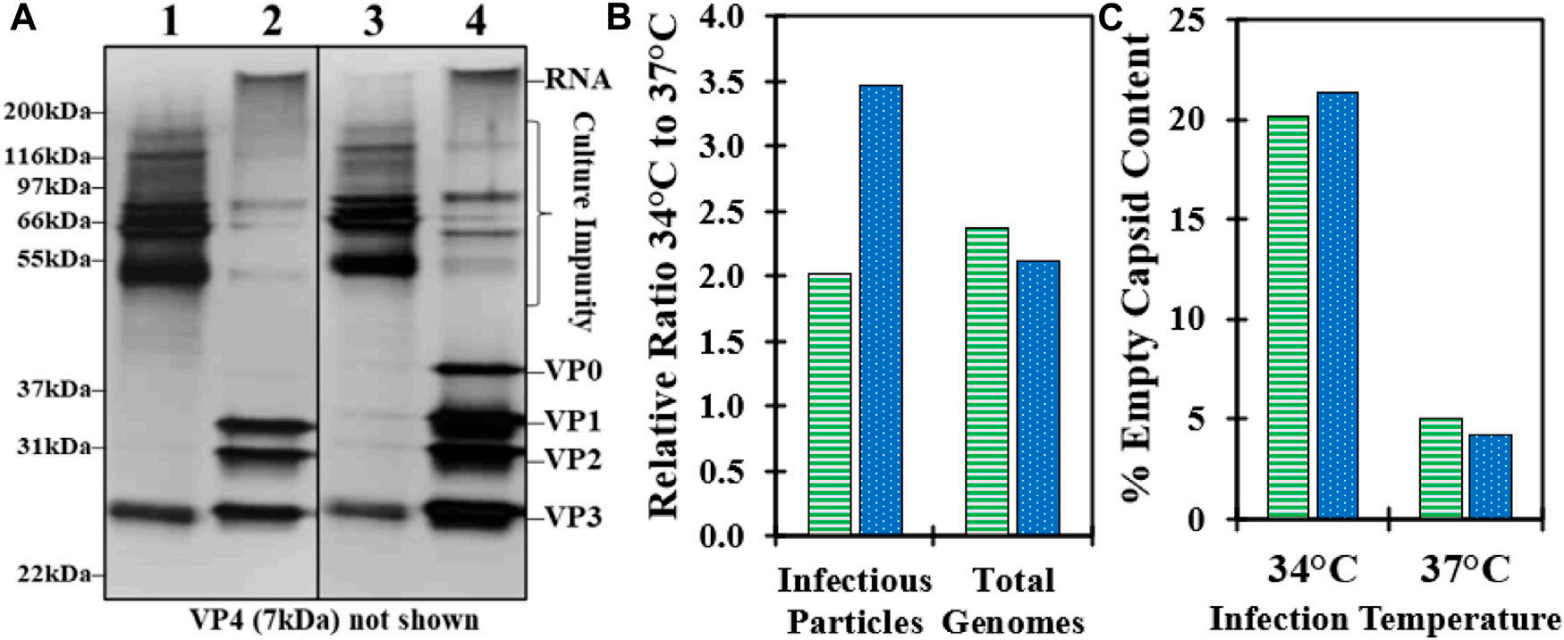
Impact of cell culture controlled at 37°C and 34°C during infection on downstream GSH affinity chromatography performance. **(A)** SDS-PAGE of CB (100x pre-dilution) and GSH elution (no pre-dilution) harvested from bioreactors controlled at 37°C (lane 1–2) and 34°C (lane 3–4) from experiment 1. **(B)** GSH elution sample relative ratio of the 34°C–37°C material from experiment 1 (green, horizontal bar) and experiment 2 (blue, dotted) for infectious particles by relative infectivity assay and total genomes by RT-qPCR. **(C)** GSH elution sample % empty capsid content by RP-UPLC for the 34°C and 37°C materials from experiment 1 (green, horizontal bar) and experiment 2 (blue, dotted).

To investigate the effect of infection temperature on downstream purification, GSH affinity chromatography was performed using harvested CB produced at both 34°C and 37°C from experiments 1 and 2. An overlay of the absorbance at 280 nm chromatograms indicated a higher concentration of the total protein in the 34°C GSH elution compared to 37°C (Supplementary Figure S3). Analysis of SDS-PAGE from previous confirmed studies reports of a VP0 band in the GSH elution in the 34°C material compared to a very faint VP0 band in the 37°C material (Figure 2A) (Swartz et al., 2022). The distribution of bands in the CB was similar and mostly consisted of serum proteins such as BSA from the cell culture media. Some of the cell culture impurity bands were detected in the GSH elution at slightly higher band intensity in the 34°C material. The GSH elution samples were further characterized by relative infectivity, viral genome RT-qPCR, and viral protein RP-UPLC assays. The GSH elution produced from 34°C infection temperature culture contained more than two-fold higher infectious particles and viral RNA genomes relative to 37°C (Figure 2B). However, the 34°C elution was composed of approximately 20% empty capsids compared to less than 5% for the 37°C sample (Figure 2C). These data demonstrated that the >two-fold increase in the CVA21 cell culture titer from 34°C infection was maintained across affinity chromatography, but an increased proportion of empty capsids co-eluted with the full, infectious particles.

### 3.3 Evaluation of GSH chromatography residence time and binding capacity

**FIGURE 3:**
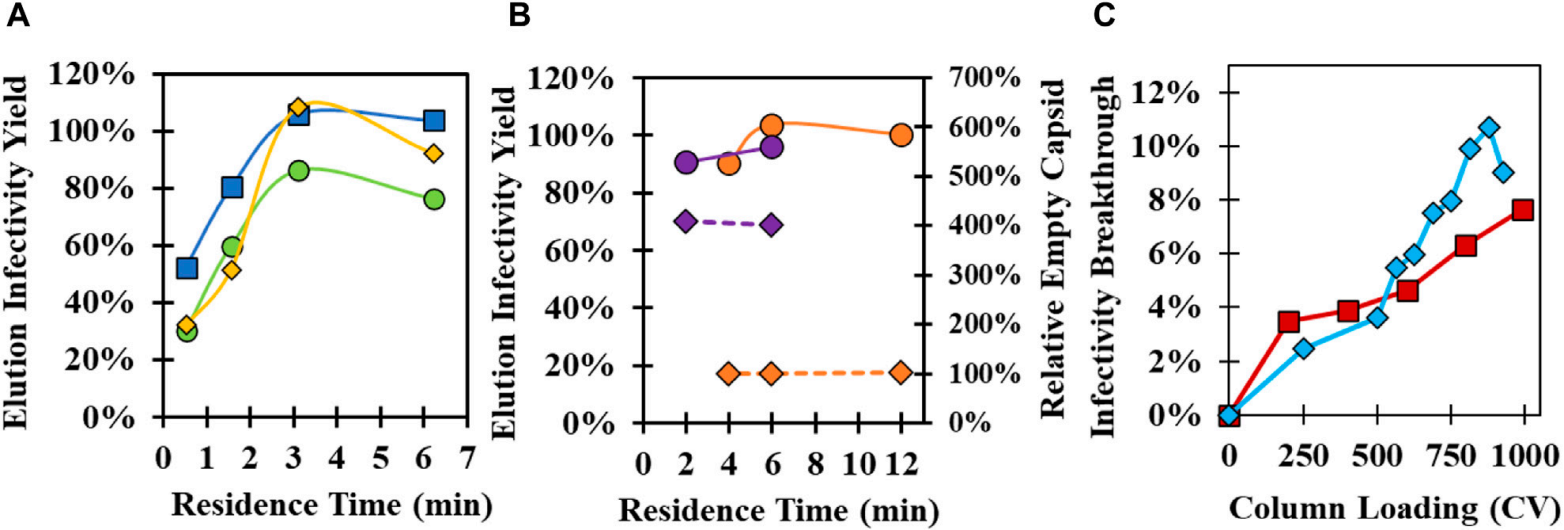
Investigation of the GSH affinity resin supplier, residence time, and binding capacity. **(A)** Evaluation of GSH elution infectivity yield at different residence times using 1 mL prepacked columns packed with Glutathione Sepharose 4 FF (blue square), Glutathione Sepharose 4B (green circle), and Pierce Glutathione (yellow diamond) with 37°C infection temperature CB. **(B)** Impact of residence time on infectivity yield (circle, solid line) and relative empty capsid content (diamond, dashed line) using 20 mL Glutathione Sepharose 4 FF columns with 37°C infection temperature CB (orange) and 34°C infection temperature CB (purple). Empty capsid content relative to the 6 min residence time 37°C infection temperature CB condition is shown. **(C)** GSH affinity chromatography breakthrough experiments using 20 mL Glutathione Sepharose 4 FF columns loaded at a 6 min residence time with 34°C infection temperature CB (red square) and concentrated and purified full capsid material (blue diamond). Purified material was diluted to the same particle concentration in CB prior to loading.

GSH affinity chromatography parameters (Supplementary Figure S1) were investigated using both 37°C and 34°C infection materials to maximize infectivity yield and to determine operational conditions that may improve empty capsid clearance in 34°C infection harvests. The effect of the residence time on the GSH elution infectivity yield was initially evaluated with three commercially available, prepacked 1 mL columns using 37°C infection harvests (Figure 3A). High infectivity yield was obtained at residence times greater than 3 min for GSH Sepharose 4 FF and Pierce Glutathione columns, while lower yields were observed across all residence times for GSH Sepharose 4B. Elution yields decreased for all columns below 3-min residence time due to losses to the GSH FT. GSH Sepharose 4 FF maintained higher yield than the other columns at faster flowrates and was selected for all future experiments. Residence time was studied using prepacked GSH Sepharose 4 FF 20 mL columns with a 10 cm bed height using 37°C and 34°C infection materials (Figure 3B). Greater than 90% yield was observed across all residence times tested, but there was a small decrease in yield at less than 6 min. Similar to previous experiments, the empty capsid content of the GSH elution from 34°C infection CB was four-fold higher than 37°C infection CB. Interestingly, there was no impact of residence time on empty capsid binding across the conditions tested (Figure 3B).

Binding capacity experiments were performed with the 20 mL GSH column using the 34°C infection CB and a previously purified CVA21 sample containing only full capsids pre-diluted in Equil buffer to the same total particle concentration as the 34°C infection CB. Infectivity breakthrough in the GSH FT was evaluated across 1,000 CV loading at a 6 min residence time. Surprisingly, only ∼8% infectivity breakthrough was detected at the end of the 1,000 CV loading for the CB sample, and the breakthrough curve overlaid with that of the pre-purified sample (Figure 3C). This demonstrated a highly specific interaction between the GSH ligand, where the binding capacity of infectious virions was not impacted by a crude matrix of cell culture lysate. Pure material was prepared at a higher concentration to achieve complete breakthrough curves, and the 10% dynamic binding capacity (DBC) was determined to be 2 × 10^14^ particles/mL resin (Supplementary Figure S4).

### 3.4 Investigation of GSH chromatography wash buffer ionic strength and pH

**FIGURE 4:**
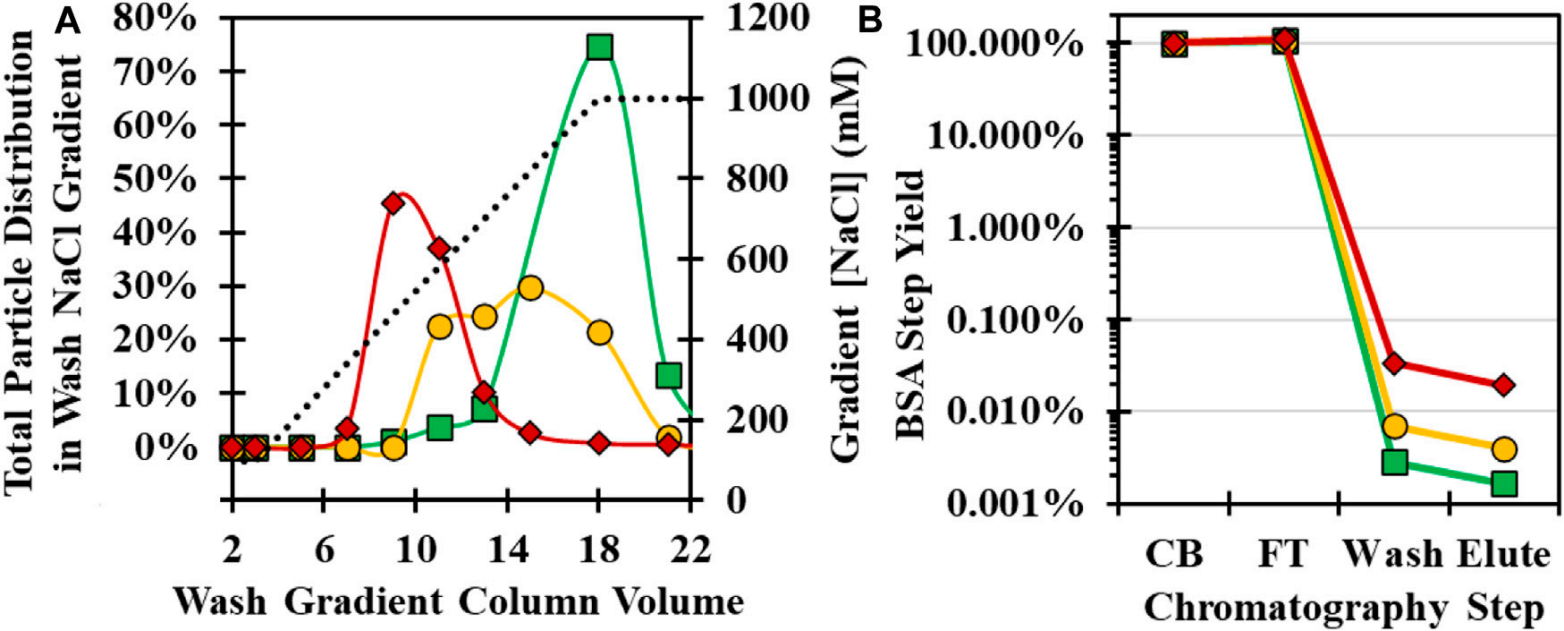
Optimization of GSH chromatography wash buffer NaCl concentration and pH conditions with 34°C infection temperature CB. **(A)** Total viral particle distribution by anti-VP1 CE Western blot in a NaCl wash gradient from 150–1,000 mM NaCl (dotted black line) at pH 7 (green square), pH 8 (yellow circle), and pH 9 (red diamond). **(B)** BSA impurity clearance by BSA ELISA across GSH chromatography steps operated at pH 7 (green square), pH 8 (yellow circle), and pH 9 (red diamond).

After loading CB, the GSH column was washed with an increased ionic strength buffer to remove non-specifically bound cell culture impurities such as BSA. The wash buffer NaCl concentration and pH were evaluated using an initial low-salt wash of 100 mM NaCl, followed by a gradient of up to 1,000 mM at pH 7, 8, and 9 using the 34°C infection material. Analysis of SDS-PAGE showed that impurities were distributed in all gradient fractions, but unexpectedly, viral protein bands were also detected within the NaCl gradient (Supplementary Figure S5A). Previously, only free GSH in the mobile phase had been demonstrated to elute CVA21 from the GSH ligand (Swartz et al., 2022). Higher pH resulted in a lower critical concentration of NaCl necessary to elute CVA21 particles, similar to cation exchange chromatographic behavior (Figure 4A). At pH 9, CVA21 was eluted at ∼300 mM NaCl, while at pH 7, CVA21 was partially eluted at 1,000 mM NaCl and in the subsequent 1 mM GSH elution buffer. In all wash gradient fractions, there did not appear to be any resolution between empty and full capsids by SDS-PAGE (Supplementary Figure S5A).

To compare impurity clearance, pooled fractions from the wash gradient and retains from the other chromatography steps were tested for BSA concentrations by ELISA assay. Greater than 99.9% of the BSA mass in the CB flowed through during loading, but at higher pH, increasing BSA was detected in the GSH wash and elution fractions (Figure 4B). A second experiment was performed with selected wash conditions of 400 mM NaCl, pH 8, and 700 mM NaCl, pH 7, in an 8-CV wash step, and similar purity was observed between the 4-CV elution samples by SDS-PAGE (Supplementary Figure S5B). Each purification run achieved >4 logs of BSA clearance from the CB with 92 ng/mL and 47 ng/mL in the GSH elution for the pH 8 and 7 wash, respectively. The pH 8 wash run had higher CVA21 elution yield and was selected as the optimal condition, but further investigation into a pH 7 wash may increase yield and purity.

### 3.5 Elution buffer conditions and demonstration of polishing chromatography

**FIGURE 5:**
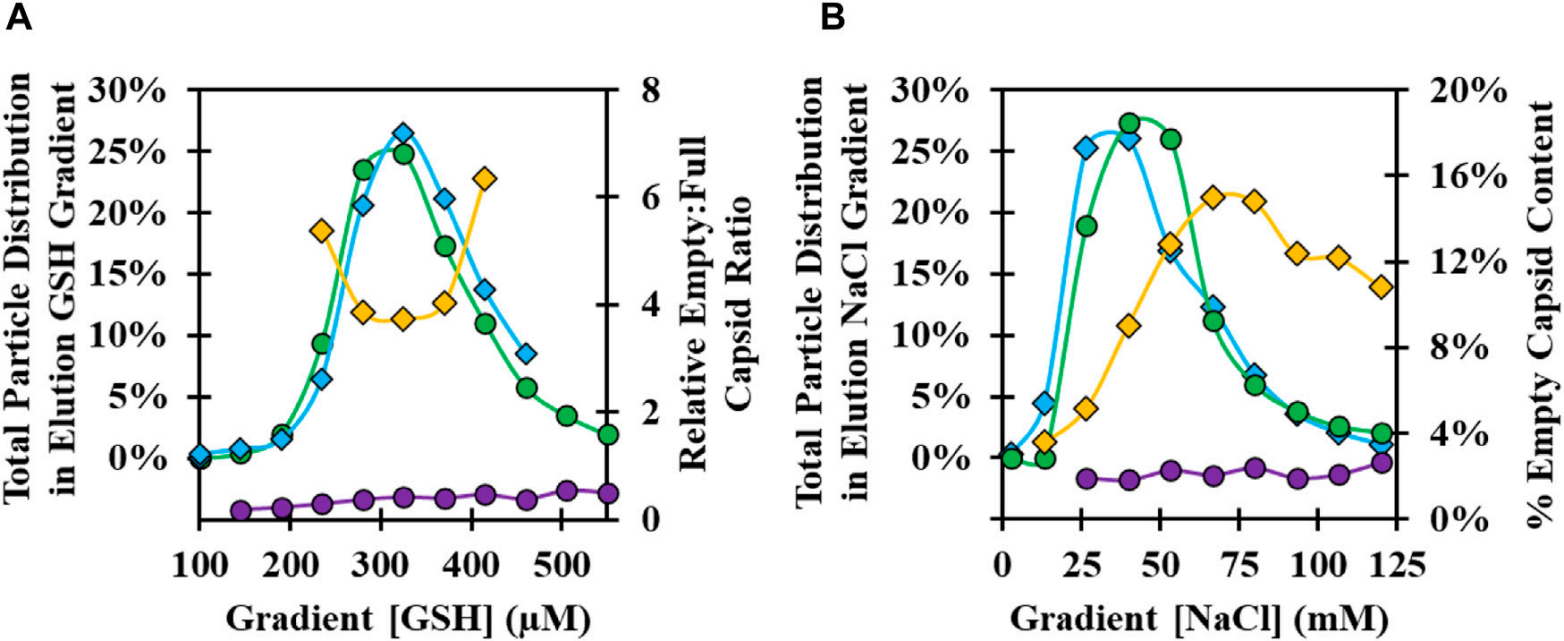
Optimization of GSH chromatography elution buffer GSH and NaCl concentration at pH 8. **(A)** Analysis of GSH elution gradient fractions from 100–600 µM GSH for 37°C infection temperature harvests: microcarrier CB produced with serum at infection, total viral particle distribution by anti-VP1 CE Western blot (green circle), and empty:full capsid ratio by anti-VP4 CE Western blot (purple circle); iCELLis CB produced without serum at infection, total viral particle distribution (blue diamond), and empty:full capsid ratio (yellow diamond). **(B)** Analysis of GSH elution gradient fractions from 0–125 mM NaCl: 37°C infection temperature material total viral particle distribution (green circle) and % empty capsid (purple circle); 34°C infection temperature material total viral particle distribution by anti-VP1 CE Western blot (blue diamond) and % empty capsid by RP-UPLC (yellow diamond).

The GSH elution buffer consists of free GSH in the mobile phase to displace the bound CVA21 virions. A concentration of only 1 mM GSH was determined to be sufficient for high recovery, but lower concentrations of GSH were investigated across a 100–1,000 µM GSH gradient to evaluate a potential separation of empty and full capsids. A GSH concentration gradient was evaluated at pH 8 with 100 mM NaCl using two types of 37°C infection harvest materials: a microcarrier harvest material produced with serum during infection, and iCELLis harvest material produced without serum during infection, where an in increased proportion of empty capsids bound to the GSH ligand. A similar distribution of the total viral particles was observed for both feed materials with a peak at about 300 µM GSH (Figure 5A). Empty capsids present in the iCELLis-produced material bound and were co-eluted across the GSH gradient with full particles, whereas empty capsids were in low abundance in the GSH gradient elution from the 37°C infection microcarrier CB (Figure 5A; Supplementary Figure S6A). There was no resolution between full and empty capsids observed across the GSH gradient, but these results confirmed that 1 mM GSH was ideal for the elution of virus particles.

Elution buffer ionic strength and pH were investigated at a fixed 1 mM GSH concentration using a NaCl elution gradient from 0–100 mM NaCl at pH 7, 8, and 9 with a microcarrier CB produced using 34°C and 37°C infections. At pH 8 and 1 mM GSH, a comparable total particle distribution was observed in the NaCl gradient with a peak at about 40 mM NaCl (Figure 5B). Unlike the GSH gradient results, there was evidence of a potential means for partial empty and full capsid separation from the 34°C material. In the beginning of the gradient at ∼25 mM NaCl, higher-purity full capsids elute first, followed by an increase in empty capsids with higher NaCl concentration. In addition, an increasing intensity of impurity bands was observed in the fractions with higher NaCl concentration and in a strip sample with 1 M NaCl (Supplementary Figure S6B). Similar trends in the viral particle distribution across the gradient were observed at pH 7–9 for both the 34°C and 37°C materials, with the viral particle elution peak occurring at higher NaCl with decreasing pH (Supplementary Figure S6C).

An elution buffer consisting of 1 mM GSH and 75 mM NaCl at pH 8 was selected as the ideal condition for maximizing CVA21 elution yield and purity. The optimized GSH affinity chromatography process was performed at the laboratory scale using the CB produced from the 34°C infection cell culture (Supplementary Figures S7A, B). Despite the aforementioned efforts to reduce empty capsids and serum proteins in the GSH elution, polishing chromatography was investigated to separate the residual impurities. Anion exchange (AEX) and cation exchange (CEX) chromatography methods were established, as described in a previous work (Konstantinidis et al., 2022). AEX of the the GSH elution was evaluated by direct loading to a POROS 50 HQ packed column, where CVA21 virions flowed through at 75 mM NaCl at pH 8 and residual serum proteins bound (Supplementary Figure S7C). However, empty capsids were still present in the AEX FT, and a CEX method was, therefore, developed using POROS 50 HS columns that efficiently separated empty capsids from full capsids. These results demonstrated a robust three-column chromatographic process using an optimized affinity capture step and two ion exchange steps to isolate highly purified, infectious CVA21 particles from high-titer microcarrier cell culture harvests controlled at 34°C during infection.

### 3.6 Scale-up of the microcarrier bioreactor and GSH chromatography processes

Laboratory-scale experiments were performed using 3 L microcarrier bioreactors, small-scale clarification filters, and 20 mL GSH chromatography columns. To accommodate clinical manufacturing requirements, CVA21 purification was scaled-up and demonstrated at 100 L and 250 L process scales across seven batches (Table 2). Cell culture was performed in 50 L or 250 L single-use bioreactors (SUBs). Agitation scaling was performed to match power input per diameter of the impeller cubed, reflective of the shear sensitivity of microcarrier-bound cells and higher shear in the area immediately surrounding the impeller. To minimize the high-energy, high-shear regime, power was distributed through an upsized impeller diameter designed for larger working volumes. This reduced agitation and power input maintained compared to a standard size impeller. Air overlay gassing was constant and scaled to approach the gas volume per unit of working liquid volume per minute (VVM). The oxygen and carbon dioxide sparging strategy was controller-driven and applied to both 50 and 250 L working volumes to maintain dissolved oxygen and pH setpoints. The temperature during infection was controlled at 37°C for batches 1–3 and changed to 34°C for batches 4–7. Clarification was performed using 0.5–1 m^2^ scale depth filters to remove microcarriers and large host cell debris. Endonuclease treatment was performed to digest HC-DNA in batches 1–2 and was removed from the process in batches 3–7. The optimized GSH affinity chromatography process was scaled-up to 0.8–1.5 L single-use, prepacked OPUS columns controlled by using an AKTA ready single-use flow path system. Relative to the 20 mL scale, key variables including column path length, residence time, and step CVs were maintained using optimized buffer conditions including a 400 mM NaCl, pH 8 wash and a 1 mM GSH, 75 mM NaCl, pH 8 elution. To demonstrate scalability, the CBs from large-scale batches 6 and 7 were also run on 20 mL GSH columns, and similar column performance and consistent infectivity yield were achieved at both process scales (Supplementary Figures S8A–C).

**FIGURE 6:**
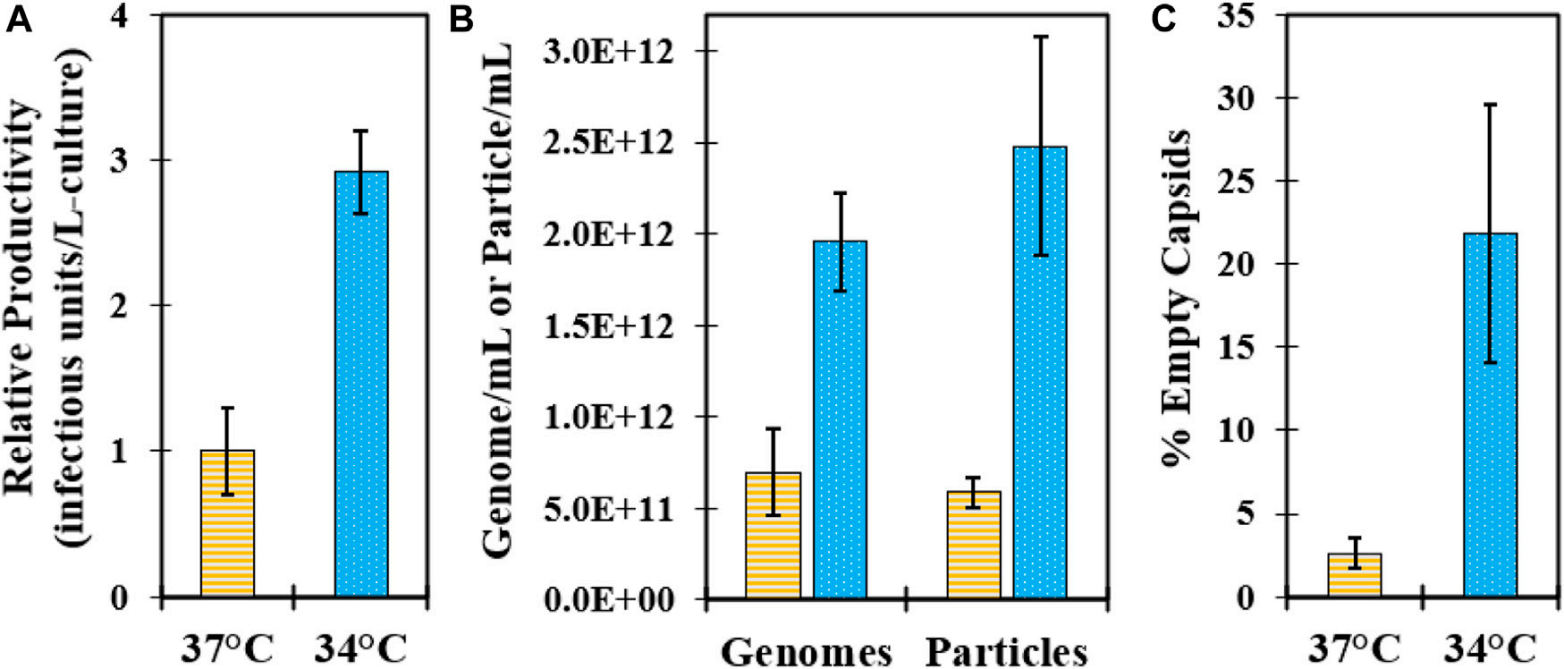
Characterization of GSH chromatography elution purified from large-scale microcarrier bioreactors controlled at 37°C (yellow, horizontal bar) and 34°C (blue, dotted) during infection. Data represent average values from batches shown in Table 2, and error bars represent one standard deviation. **(A)** Relative comparison of batch productivity at GSH elution. **(B)** Comparison of viral genomes by RT-qPCR and viral particles by VP1 CE Western assays at GSH elution. **(C)** Comparison of % empty capsids by RP-UPLC in the GSH elution.

Key attributes of the GSH elution were evaluated across large-scale batches produced from 37°C to 34°C infection temperatures. Excellent GSH elution infectivity yield was maintained across all batches ranging from ∼85–100% (Table 2), and the average productivity of 34°C batches in the GSH elution was about three-fold higher than that of the 37°C batches, consistent with the laboratory-scale evaluation (Figure 6A). There was a similar increase in the total genomes, but more than a four-fold increase in total particles was detected in the 34°C batch elution (Figure 6B). The increase in total particles was attributed to the increased proportion of empty capsids present in the GSH elution from 34°C harvests (Figure 6C). Efficient impurity clearance was demonstrated across all large-scale GSH chromatography runs. Cell culture harvests contained approximately 500,000 ng/mL BSA that was cleared to a range of about 10–100 ng/mL in the GSH elution, representing greater than 5 logs of the overall process clearance (Table 2). Residual HC-DNA at harvest ranged from 1,000–2000 ng/mL and was reduced by the two-step clarification scheme and by digestion by an endonuclease in batches 1–2. However, the endonuclease treatment was removed from the process after batch 2 as GSH chromatography cleared HC-DNA to almost undetectable levels even without endonuclease treatment (Table 2). These results demonstrate an efficient scale-up from 3 L to 250 L microcarrier bioreactors and from 20 mL to 1.5 L GSH chromatography columns and demonstrate the significant increase in productivity in GSH elution from 34°C harvests.

## 4 Discussion

Oncolytic CVA21 drug substance was manufactured for early clinical supply using poorly scalable technologies including MRC-5 static culture in cell stacks and purification by ultracentrifugation. Similar to the early production of other viral vectors [26], a scale-out approach was employed with dozens of 10-layer cell stacks and several rounds of ultracentrifugation over multiple days. To support the requirements of late-stage clinical production and commercialization, microcarrier-stirred tank bioreactors and affinity chromatography were investigated as scalable solutions. A process using laboratory-scale 3 L bioreactors initially controlled at 37°C during infection and 20 mL column GSH affinity chromatography columns was established previously, where the empty capsids present in cell culture harvests did not bind to the GSH ligand, and excellent full capsid purity was obtained in the GSH elution (Swartz et al., 2022). However, it was discovered that certain cell culture conditions such as the infection temperature setpoint or absence of serum during infection resulted in increased empty capsid binding during loading and decreased GSH elution full capsid purity. To increase purification process robustness and maintain high full capsid purity, a two-staged strategy was implemented in this work. First, empty capsid clearance was investigated from different cell culture harvests through optimization of GSH chromatography parameters, and if insufficient resolution is observed, the subsequent polishing chromatography steps are evaluated.

**SCHEME 1:**
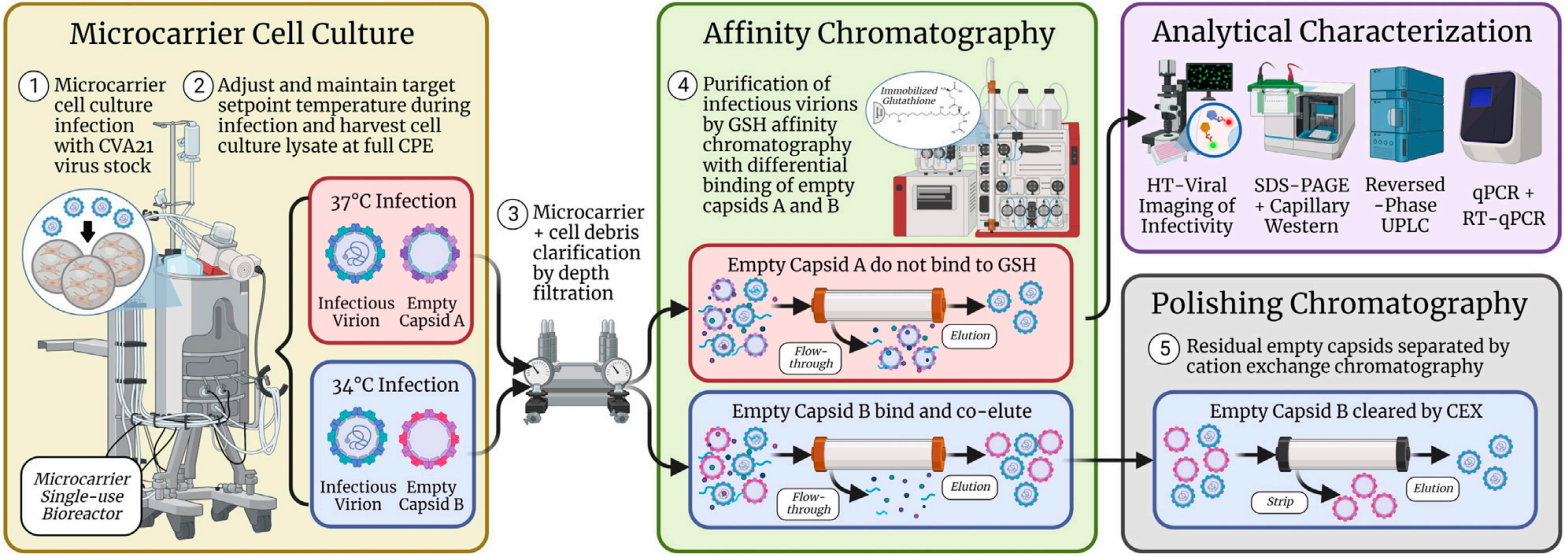
Microcarrier cell culture infected with CVA21 at 37°C produced empty capsids (empty capsid A) that were separated from full, infectious virions across GSH affinity chromatography. However, cell culture infected at 34°C generated empty capsids (empty capsid B) that bound and co-purified with infectious virions such that a polishing chromatography step using CEX was implemented to clear the residual empty capsids from the GSH elution. CVA21 virus particles were characterized by several assays including high-throughput viral infectivity, SDS-PAGE, CE Western, RP-UPLC, and qPCR.

Prior to the GSH chromatography studies, the cell culture temperature setpoint during infection was studied to maximize the infectious titer. Three sets of bioreactor experiments were performed, and an optimal temperature setpoint of 34°C resulted in a two–three-fold increase in peak infectivity relative to the 37°C condition (Figure 1; Supplementary Figure S2). This was the first demonstration of this finding for CVA21 and is consistent with reports of ideal enterovirus cell culture propagation at sub-physiological temperatures (Yoshii et al., 1977; Ashraf et al., 2013; Chen et al., 2021). One explanation may be that viruses known to infect the upper respiratory tract replicate more efficiently at the lower temperatures present in the upper airway (Papadopoulos et al., 1999). This higher cell culture productivity at 34°C was maintained across GSH chromatography purification, but about 20% of the viral particles in the GSH elution were empty capsids compared to <5% from the 37°C condition (Figure 2). Although harvests from both 34°C and 37°C infections contained a similar proportion of empty and full particles, cell culture conditions that are more permissive to viral replication resulted in the production of empty capsids with increased GSH binding (Swartz et al., 2022). Because infectious particle yield in the GSH elution remained high across all conditions tested, only empty capsid–GSH binding appeared to be impacted by the different temperatures during infection. Based on these observations, empty capsids produced from the cell culture infected at 37°C (empty capsid A, Scheme 1) were sufficiently removed in the GSH FT and did not require further optimization of GSH chromatography parameters for additional clearance. Conversely, empty capsids produced from the cell culture infected at 34°C (empty capsid B, Scheme 1) co-eluted with infectious CVA21 virions and required either further optimization of GSH chromatography parameters or development of a polishing chromatography step to clear residual empty capsids.

GSH chromatography parameters were evaluated to determine whether empty capsids could be cleared from the 34°C infection CB. Residence time (Figure 3B), wash buffer NaCl concentration (Figure 4A), and elution buffer GSH concentration (Figure 5A) did not demonstrate any detectable separation between the empty and full capsids. NaCl gradient elution at 1 mM GSH (Figure 5B) studies indicated that some full particle enrichment may be possible. At lower NaCl elution concentrations, full capsids may be preferentially displaced by free GSH in the elution buffer prior to empty capsids, but further development would be necessary to increase resolution. As an alternative, polishing chromatography using CEX was established as an effective means of empty capsid separation with highly infectious particle yield (Supplementary Figure S7) (Konstantinidis et al., 2022). Purification of the high-titer 34°C infection temperature harvests with GSH affinity combined with polishing chromatography was selected as the ideal strategy to increase productivity while maintaining high full capsid purity (Scheme 1).

Several benefits of the established GSH affinity chromatography method were demonstrated in this work. First, GSH chromatography resin is commercially available from many suppliers for the purification of GST-tagged recombinant proteins and is less expensive than other affinity resins (Schäfer et al., 2015). This investigation, along with a previous study (Swartz et al., 2022), was the first known demonstration of the use of immobilized GSH as an affinity ligand for a non-GST-tagged virus particle. Three off-the-shelf, 1 mL prepacked columns were screened, and all obtained ∼80% or higher infectivity yield at residence times of more than 3 min (Figure 3A), demonstrating that other available immobilized GSH resins should function similarly. Second, a high-dynamic binding capacity of 2E+14 particles/mL-resin was demonstrated (Supplementary Figure S4), equivalent or better than the capacity of commercial AAV affinity resins (Terova et al., 2018). In addition, similar infectivity breakthrough profiles were observed across 1,000 CVs of loaded CB and purified material, indicating highly selective binding even in a crude, serum-containing feed stream (Figure 3C). Targeting such a high capacity for CB loading is not attainable in a reasonable timeframe using a 6 min residence time, but application of in-line concentration with single-pass tangential flow filtration could help maximize resin utilization.

A third benefit of GSH chromatography is a low-salt and neutral pH elution enabled by displacement with free GSH in the mobile phase. Many affinity chromatography elution buffers require acidic pH or high salt (Zhao et al., 2019), but for GSH affinity chromatography, a high-yielding elution was achieved with just 75 mM NaCl and 1 mM GSH at pH 8 (Figure 5B). The low GSH concentration necessary for CVA21 displacement may also limit a co-elution of potentially bound host cell GST enzymes present in cell culture harvests, which may require as high as 10 mM GSH for elution (Harper and Speicher, 2011). Furthermore, cation exchange behavior was observed in both wash (Figure 4A) and elution NaCl gradient (Supplementary Figure S6C) studies where CVA21 particles eluted at lower ionic strengths with increasing pH. This was surprising considering the theoretical isoelectric point based on the amino acid sequence of the capsid structural proteins is 6.1 (Newcombe et al., 2003). However, the accessible surface charge distribution within the GSH binding site of the assembled capsid may be net-positive at neutral pH. Several native enteroviruses are known to interact with intracellular GSH during capsid assembly through binding to a conserved positively charged region within a protomer–protomer pocket (Duyvesteyn et al., 2020; Bahar et al., 2022). The GSH chromatography ligand is immobilized to the stationary phase by the central cysteine sulfhydryl group through a linker but maintains the same net electrostatic charge as with intracellular GSH at physiological pH (2 positive and 1 negative) (Tummanapelli and Vasudevan, 2015). Furthermore, the 75 mM NaCl, pH 8 GSH elution allowed for a direct, low-salt loading to AEX polishing chromatography that bound the residual protein and HC-DNA impurities while CVA21 flowed through (Supplementary Figure S7). Direct loading is ideal for operational efficiency because it eliminates the need for a buffer adjustment and mixing step. Lastly, the GSH column can be regenerated and reused over multiple cycles without loss of functionality (Supplementary Figure S9).

The final objective of this work was to demonstrate scalability of the established CVA21 production process from 3 L to 250 L process scales. Consistent chromatography performance and yield were maintained between the 20 mL and 1.5 L GSH column purification methods (Supplementary Figure S8). On a large scale, higher GSH elution productivity, viral genomes, and total particles were observed in 34°C batches along with 5–10-fold higher % empty capsids (Figure 6). These residual empty capsids (empty capsid B) were cleared by CEX polishing chromatography to a similar level as the GSH elution from 37°C batches, with only a faint VP0 band detectable by SDS-PAGE (Supplementary Figure S7C). Excellent infectivity yield and impurity clearance were obtained across all batches, and the removal of endonuclease treatment from the process in batches 3–7 resulted in significant cost savings. Compared to early scaled-out clinical production methods, operational efficiency and throughput were improved using the 250 L SUB system and AKTA ready skid to process more than 10-fold the volume.

## 5 Conclusion

This work presented a productive and scalable solution for the manufacture of an oncolytic virus using a challenging adherent cell line grown in serum-containing media. Static cell culture and ultracentrifugation methods were redeveloped with microcarriers in stirred-tank bioreactors and glutathione affinity chromatography using single-use, disposable technologies. Key process parameters were investigated to maximize viral productivity and purity. Lowering the cell culture temperature during infection to 34°C from 37°C resulted in a two–three-fold increase in infectivity but resulted in the co-elution of empty capsids during GSH chromatography. Loading, wash, and elution chromatographic parameters were investigated to separate empty and full capsids and increase purity. Decreased elution NaCl concentration was identified as a potential means of separation, but additional work is necessary to improve the resolution. In addition, higher-wash NaCl concentration at pH 8 and decreased NaCl concentration with 1 mM GSH at pH 8 resulted in improved serum protein clearance. The production process was scaled-up by two orders of magnitude and demonstrated the increased productivity at the 34°C infection condition with excellent BSA and HC-DNA clearance across all seven batches, even after the removal of an endonuclease step. Polishing AEX and CEX chromatography processes were implemented to clear the trace residual impurities and empty capsids in the GSH elution from 34°C harvests. Future work will focus on understanding the structural differences of empty capsids produced from 34°C to 37°C infections and further characterization of the differential GSH binding. The established microcarrier bioreactor and three-column chromatography process will be evaluated with other enteroviruses and vectors to demonstrate a robust platform for the generation of high-purity, full viral capsids.

## Data Availability

The raw data supporting the conclusion of this article will be made available by the authors, without undue reservation.
