# Supplementary material for: Glutathione affinity chromatography for the scalable purification of an oncolytic virus immunotherapy from microcarrier cell culture
Source: Front Bioeng Biotechnol. 2023 Jun 15;11:1193454. doi: 10.3389/fbioe.2023.1193454 (PMC10310922; doi:10.3389/fbioe.2023.1193454)
Supplement: Supplementary file 1 [file DataSheet1.docx]

*Supplementary Material*

**Supplementary Figures**


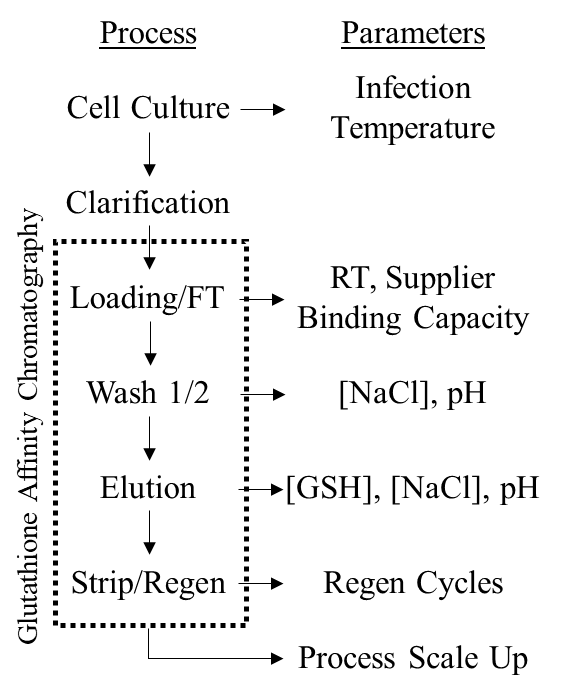


**Supplementary Figure S1**. Overview of cell culture, clarification, and glutathione affinity chromatography steps and process parameters investigated in the study.


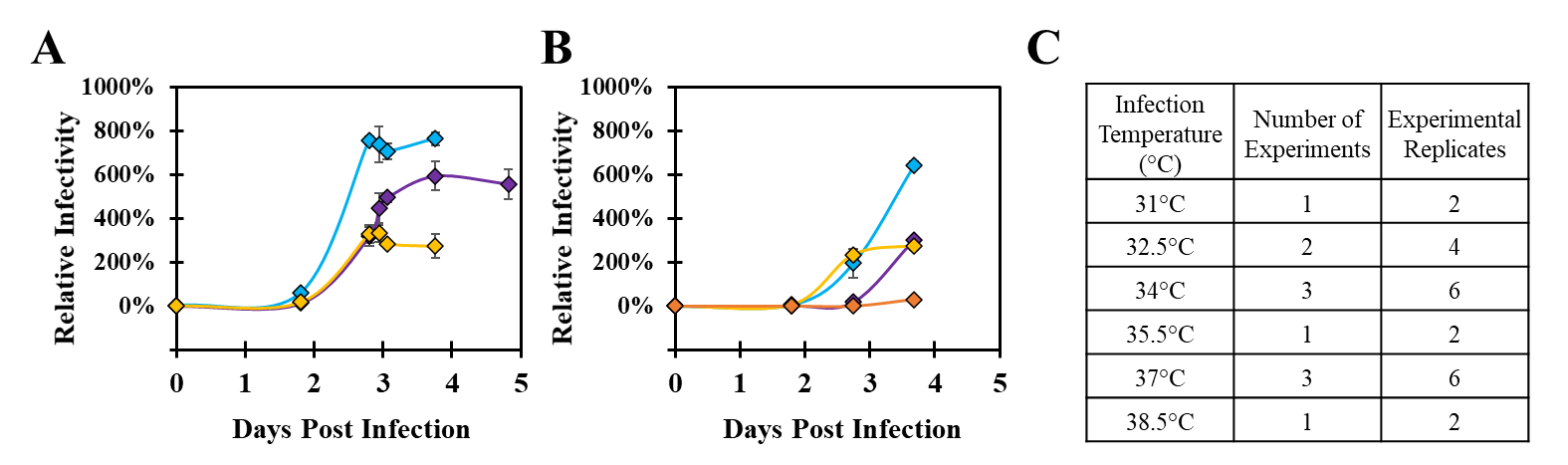


**Supplementary Figure S2**. A) Relative infectivity time course for Experiment 2 in 3 L bioreactors with infection controlled at 37°C (yellow), 34°C (blue) and 32.5°C (purple). Error bars represent one standard deviation of an average of two replicate conditions. B) Relative infectivity time course for Experiment 3 in Ambr®250HT with infection controlled at 37°C (yellow), 34°C (blue), 32.5°C (purple), and 31°C (orange). Error bars represent one standard deviation of an average of two replicate conditions. C) Summary of bioreactor experiments and experimental replicates at each infection temperature. Average results at each temperature represented in Figure 1B.

**
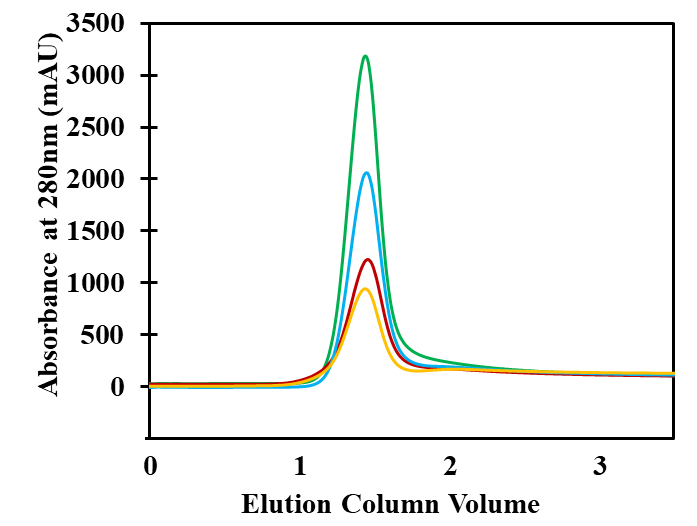
**

**Supplementary Figure S3.** Chromatogram absorbance at 280 nm snapshot of the GSH chromatography elution for Experiment 1 using 34°C infection CB (blue) and 37°C infection CB (yellow) and Experiment 2 using 34°C infection CB (green) and 37°C infection CB (red).


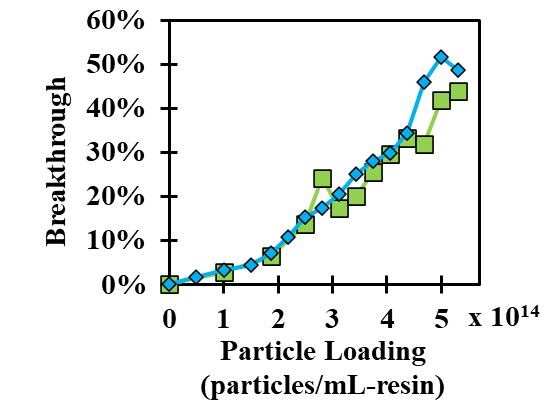


**Supplementary Figure S4.** CVA21 binding capacity on a 20 mL Glutathione Sepharose 4FF column operated at a 6 min residence time using concentrated and purified full capsid material. Data presented as infectivity breakthrough (blue diamond) and total particle breakthrough by anti-VP1 CE western (green square) as a function of total particle loading.


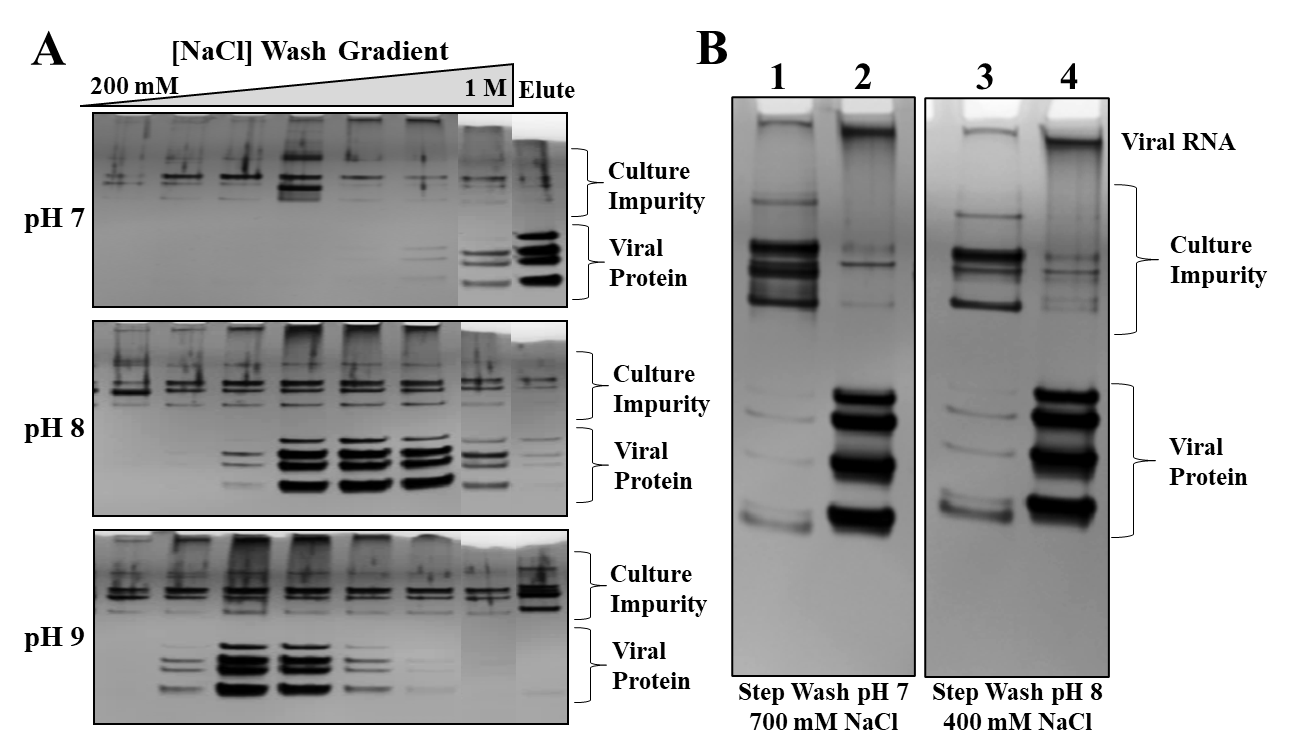


**Supplementary Figure S5.** A) SDS-PAGE of GSH chromatography wash NaCl concentration gradient fractions from 200-1000 mM NaCl at pH 7, 8, and 9 and the subsequent GSH elution fraction using 34° infection temperature CB. Cell culture impurity and viral bands indicated. B) GSH chromatography with selected step wash conditions at 700 mM NaCl, pH 7 (Lane 1) and 400 mM NaCl, pH 8 (Lane 3). The same step elution (Lane 2 and Lane 4) was performed for both arms using a buffer with 1 mM GSH at pH 8.


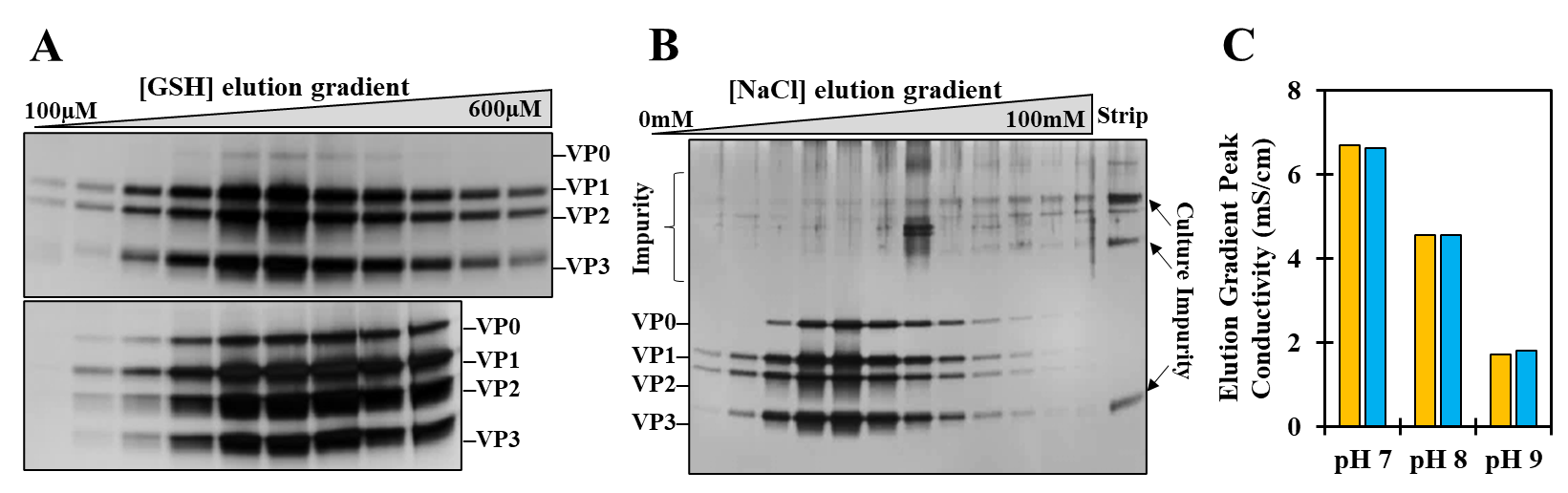


**Supplementary Figure S6.** A) SDS-PAGE of elution GSH concentration gradient fractions from 100-600 µM GSH at pH 8 from 37°C infection temperature CB (top) and from iCellis reactor CB (bottom) as described in Figure 5A. B) SDS-PAGE of elution NaCl concentration gradient fractions from 0-100 mM NaCl with 1 mM GSH at pH 8 from 34°C infection temperature CB as described in Figure 5B. C) Chromatogram analysis of the conductivity at peak absorbance at 280 nm for the elution NaCl concentration gradients with 1 mM GSH at pH 7, 8, and 9 from 37°C infection temperature CB (yellow) and 34°C infection temperature CB (blue).


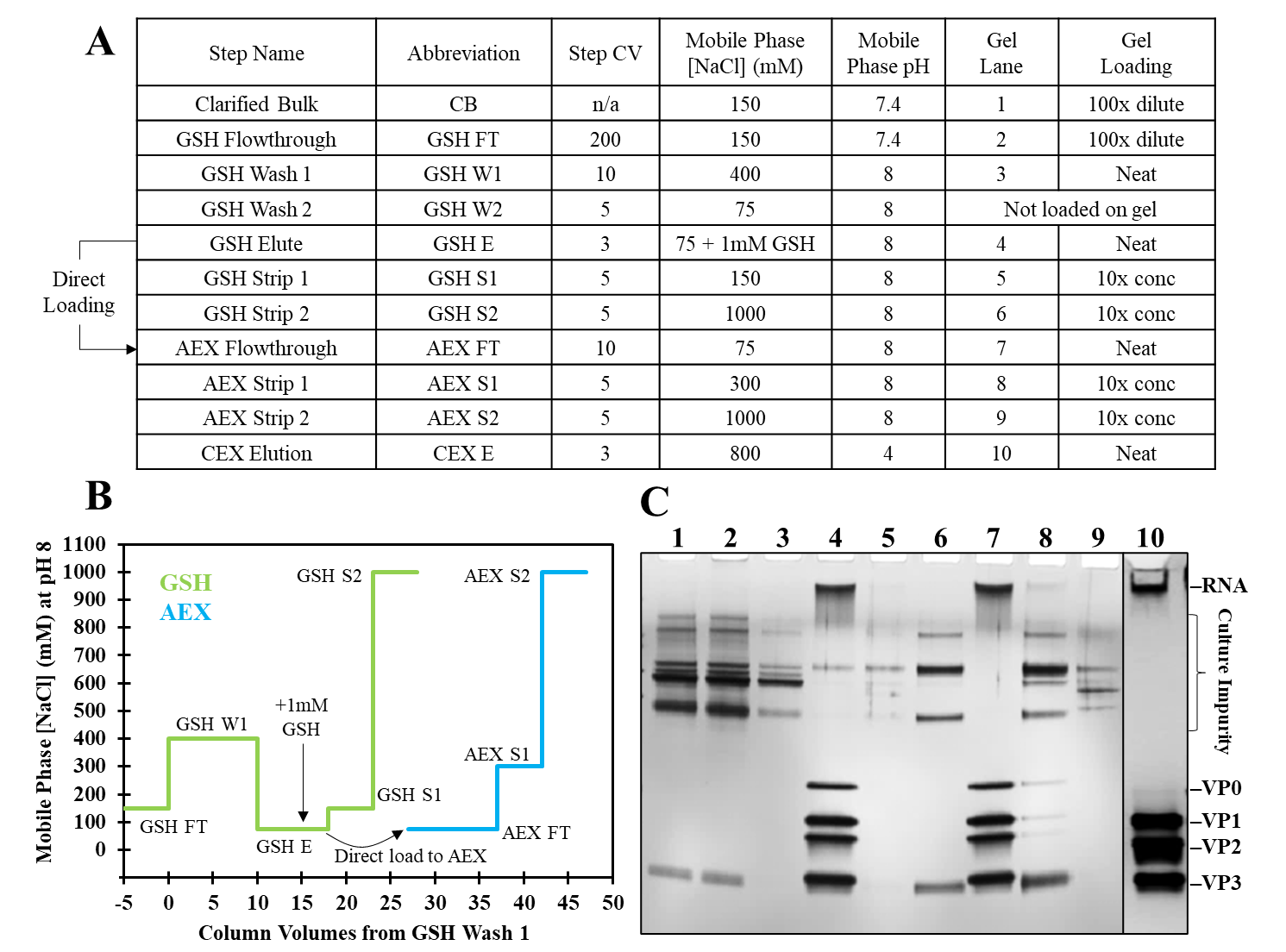


**Supplementary Figure S7.** Demonstration of GSH affinity chromatography with optimal conditions and polishing AEX and CEX chromatography from cell culture harvests using 34°C infection temperature at lab scale. A) Process step description, chromatography step column volume, mobile phase composition, and gel lane loading information shown in Supplementary Figure 7C. CB and GSH FT samples were diluted 100x with water. 10x concentrated samples were concentrated using 10 kDa Vivaspin 500 centrifugal concentrators (Sartorius AG). B) Representation of mobile phase NaCl concentration at pH 8 across GSH and AEX steps, illustrating the direct loading of the GSH elution to the AEX FT step at 75mM NaCl. C) SDS-PAGE of GSH, AEX, and CEX chromatography steps described in Supplementary Figure S7A.


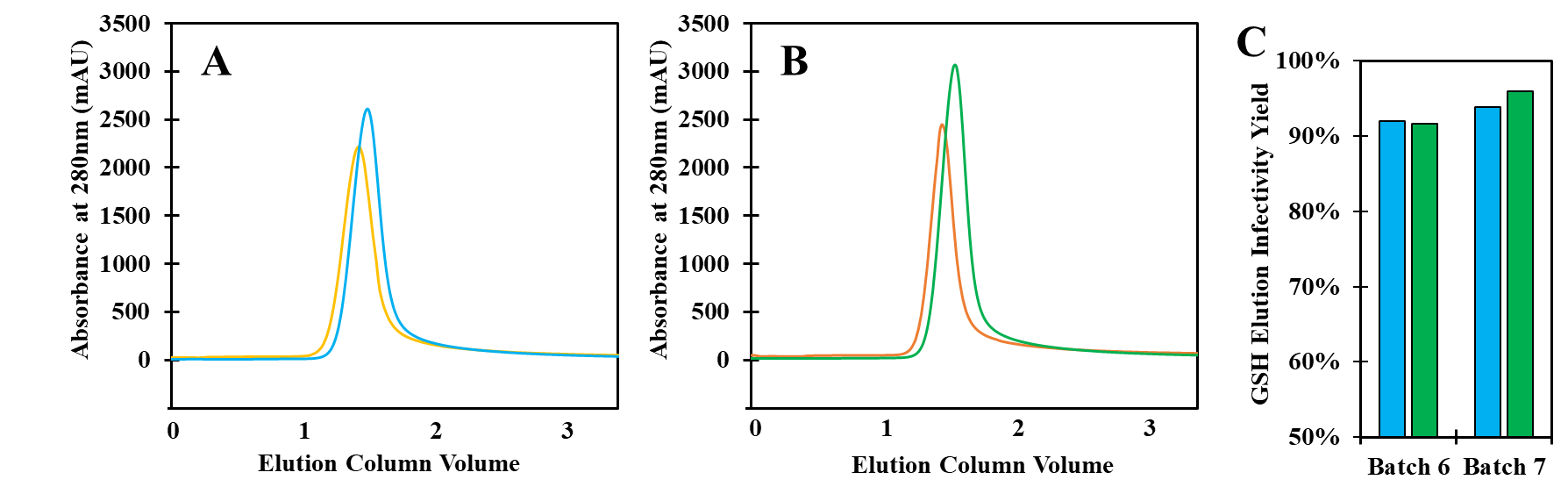


**Supplementary Figure S8.** Comparison of large scale GSH with 20 mL scale down for Batches 6 and 7. A) Batch 6 chromatogram absorbance at 280 nm during the GSH chromatography elution at large-scale with a loading of 150mL/mL-resin (yellow) and lab scale-down with a loading of 170 mL/mL-resin (blue). B) Batch 7 chromatogram absorbance at 280 nm during the GSH chromatography elution at large-scale with a loading of 150mL/mL-resin (orange) and scale down with a loading of 180 mL/mL-resin (green). C) Infectivity yield of the GSH elution for large scale columns (blue) and lab scale-down columns (green).


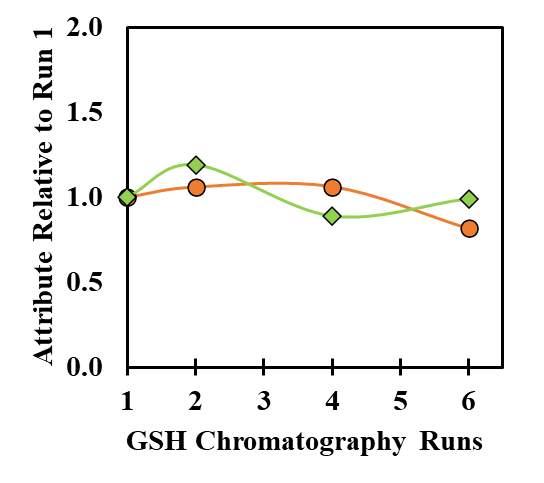


**Supplementary Figure S9.** Evaluation of GSH column regeneration with 0.1N NaOH, 1M NaCl solution and reuse across five regenerations on subsequent runs 2-6 relative to run 1. GSH elution infectivity (green diamond) and GSH elution viral particles by anti-VP1 CE western (orange circle) represented relative to the attribute in the first GSH chromatography run elution.
